# Practical Guide to Trapping *Peromyscus leucopus* (Rodentia: Cricetidae) and *Peromyscus maniculatus* for Vector and Vector-Borne Pathogen Surveillance and Ecology

**DOI:** 10.1093/jisesa/ieaa028

**Published:** 2020-11-02

**Authors:** Erika T Machtinger, Scott C Williams

**Affiliations:** 1 Department of Entomology, 4 Chemical Ecology Laboratory, Pennsylvania State University, University Park, PA; 2 Center for Vector Biology and Zoonotic Diseases, The Connecticut Agricultural Experiment Station, New Haven, CT

**Keywords:** blacklegged tick, *Ixodes scapularis*, Lyme disease, small mammals, trapping

## Abstract

Arthropods pests are most frequently associated with both plants and vertebrate animals. Ticks, in particular the blacklegged ticks *Ixodes scapularis* Say and *Ixodes pacificus* Cooley & Kohls (Acari: Ixodidae), are associated with wildlife hosts and are the primary vectors of Lyme disease, the most frequently reported vector-borne disease in the United States. Immature blacklegged ticks in the eastern United States frequently use small mammals from the genus *Peromyscus* as hosts. These mice are competent reservoirs for *Borrelia burgdorferi,* the causative agent of Lyme disease, as well as other tick-borne pathogens. To conduct surveillance on immature ticks and pathogen circulation in hosts, capture and handling of these small mammals is required. While protocols for rearing and pest surveillance on plants are common, there are very few protocols aimed at entomologists to conduct research on vertebrate–arthropod relationships. The goal of this manuscript is to provide a practical template for trapping *Peromyscus* spp. for vector and vector-borne pathogen surveillance and ecology for professionals that may not have a background in wildlife research. Important considerations are highlighted when targeting *P. leucopus* Rafinesque and *P. maniculatus* Wagner. Specifically, for tick and tick-borne disease-related projects, materials that may be required are suggested and references and other resources for researchers beginning a trapping study are provided.

The most frequently reported vector-borne diseases in the United States are tick-borne (Rosenberg et al. 2018). Among the most prevalent tick-borne diseases are Lyme borreliosis, human granulocytic anaplasmosis, babesiosis, and Powassan virus encephalitis. These diseases are caused by pathogens transmitted by two important ticks in the United States, blacklegged ticks (*Ixodes scapularis* Say), and western blacklegged ticks (*Ixodes pacificus* Cooley & Kohls) (Acari: Ixodidae).

Tick-borne pathogens are zoonotic with natural circulation in wildlife reservoirs. Mice in the genus *Peromyscus* are considered important hosts for both *I. scapularis* immature life stages. While there are over 50 extant species of *Peromyscus* in North America, white-footed mice (*Peromyscus leucopus* Rafinesque) and deer mice (*Peromyscus maniculatus* Wagner) are two of the most widely distributed and frequently captured in most areas of the United States (herein referred to as *Peromyscus* spp.) where pathogens and ticks are also found (Fig. 1). These two species have been documented to be competent reservoirs for many important tick-borne pathogens that can affect humans and animals, and in some cases, have greater competency than other small mammals and birds (LoGiudice et al. 2003, Keesing et al. 2012).

**Fig. 1.**
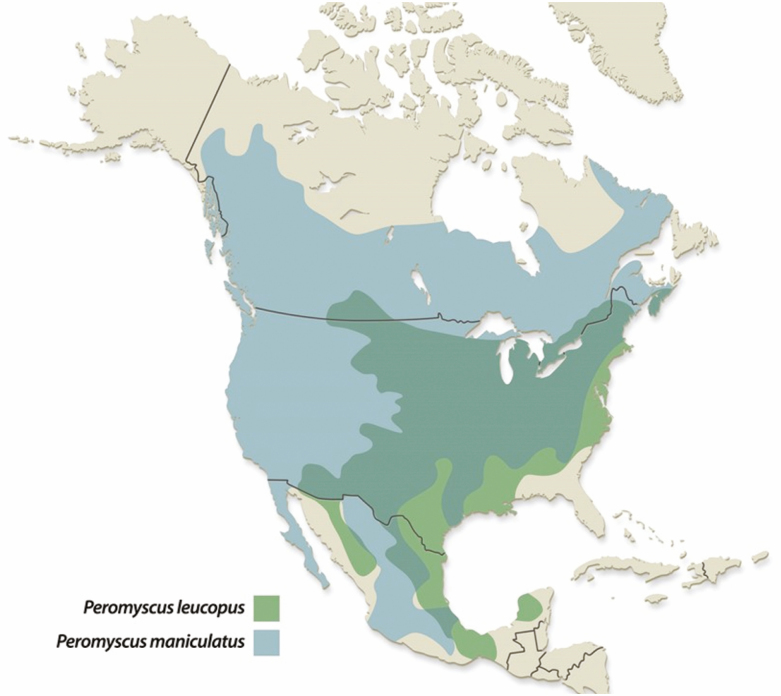
Range and overlap of *Peromyscus leucopus* and *Peromyscus maniculatus* in the United States. Drawing by Nick Sloff.

Because of their role in the epidemiology of many tick-borne diseases, live trapping *Peromyscus* spp. can provide useful tick surveillance information. While trapping *Peromyscus* spp. to evaluate tick specimens may be more labor intensive than drag sampling, parasitizing ticks removed from captured rodents can provide information on the abundance and/or presence of ticks as well as tick species, life stage, and pathogen infection in both vectors and reservoirs. The Centers for Disease Control and Prevention (CDC) has published guidance on the use of host trapping as a means of surveillance for *I. scapularis* (Eisen et al. 2018) and has suggested that these data can be used to 1) classify county status, 2) identify presence, but not prevalence of pathogens in all active life stages of ticks, and 3) document host-seeking phenology.

Beyond surveillance, trapping *Peromyscus* spp. can also provide information on tick and host ecology. Important information including tick associations with hosts, distribution in the environment, response to environmental variables, prevalence of various pathogen infection, and other factors can be obtained from trapping *Peromyscus* spp. Tick response to landscape or host-targeted control methods can also be assessed with host trapping as well as pathogen burdens and effects of these pathogens on hosts.

Historically, entomologists were very familiar with plant–insect interactions and plant rearing or inspection protocols for pests, but it is becoming increasingly important to understand vertebrate–arthropod interactions in the fields of medical and veterinary entomology as well. Many entomologists may find using vertebrates in research daunting if they have not been formally trained in animal science or wildlife biology. However, decades of previous studies allow for robust trapping methodology that, with some knowledge and resources, can be implemented with success.

The purpose of this protocol was not to provide a comprehensive review of trapping for *Peromyscus* spp. as many resources have been developed that detail ecological and practical considerations for trapping small mammals. Instead, the purpose is to provide a brief review and practical template for trapping *Peromyscus* spp. for vector and vector-borne disease surveillance and ecology based on our experience and provided for entomologists or biologists that may not have a background in wildlife research. We will highlight important considerations when targeting both *Peromyscus* species, specifically for tick and tick-borne disease-related projects, suggest materials that may be required, and provide references and other resources for researchers first instituting a trapping protocol. The methods provided here represent those that have been tested and proven by the authors to be successful but are not an indication that these are the only acceptable methods. It is important to recognize that there are likely many methods that are not included that are research or researcher specific and it is expected that users of the methods provided herein will likely adapt these methods to meet their own trapping goals. In many cases, recommendations are given that may require the reader to consult additional reference material and it is assumed that readers may supplement this protocol with additional references as required based on their individual understanding of wildlife biology and management. To that end, this is also not an exhaustive list of methods for trapping all small mammals and resources for considerations not highlighted here will be provided. All photographs were taken from research conducted under approved United States Department of Agriculture or Pennsylvania State University Institutional Animal Use and Care Committee Protocols.

## Experimental Design

### What Permits, Protocols, and Personal Protective Equipment May Be Required?

Prior to trapping, safety risks should be addressed and all permissions, permits, and approved protocols received. There are some specific safety risks to consider such as in some areas of the country, hantavirus is a concern when handling rodents. In other areas, snakes, ground-dwelling wasps, or venomous spiders such as black widows (*Latrodectus* spp. Walckenaer [Araneae: Theridiidae]) may be found in traps. Appropriate precautions should be addressed with your institution based on your specific region.

Appropriate permissions including permits if on local, state, federal, or tribal lands or permissions if on private land will be required as well as an approved Institutional Animal Care and Use Committee (IACUC) protocol which will be required for publication. Permit and protocol requirements will differ by state and institution. However, in general, a methodology and experimental design will be required. If a target site does not have an obvious identifiable owner, often county or local townships will have an online GIS system that can be used to identify the parcel owner.

While in the field, researchers should wear long sleeves and pants both while setting traps and processing. Wide brimmed hats, sunscreen, and CDC-recommended insect repellent are also suggested. The use of long sleeves and pants and repellents can help protect against questing ticks and from thorns, biting insects, and poisonous plants. Use of repellent will depend on experimental design as repellents may deter ticks and odors may influence trap captures, but pyrethroid impregnated clothing or repellents with nominal odors can be used. The Environmental Protection Agency has developed a repellent search tool that may be useful in making repellent decisions (EPA 2019). Latex or nitrile gloves should be worn at all times when mouse trapping to protect researchers and hand sanitizer should be available and used frequently when gloves are removed. The passenger compartment of vehicles should be ‘mouse-free zones’ where extra clothing, water bottles, and food should be stored. Mouse traps and supplies should be transported in the open air in the back of a pickup truck where researchers are not exposed to them.

During all field work, personnel should have access to copies of all permissions, permits, and protocols, a first aid kit, and contact information for emergency services. A field safety plan should be implemented, and personnel should be trained yearly on risk mitigation and emergency procedures. Individual institutions may have guidelines for implementing these plans, but examples can be found at other institutions (Duke University 2015). While not exhaustive, Table 1 lists additional personal protective equipment for consideration. In some areas of the country, hantavirus is an important consideration and trappers should refer to CDC guidelines (Mills et al. 1995 and their health and safety groups within their organization for guidance.

**Table 1.** Personal protection supplies examples and estimated cost for *Peromyscus* spp. trapping^1^

Item	Purpose	Example	Estimated cost
First aid kit	Basic field first aid for minor injuries	Various options available	Varies
Hiking boots without laces	Aid in traversing difficult terrain. Boots without laces reduce access points ticks have to feed and legs.	Ariat Terrain pull-on boot (Ariat Footware)	$150.00
Snake gaiters	In some areas of the country, venomous snakes may be a concern. Snake gaiters can provide protection from snake bite risk.	TrueTimber Snake Gaiter (BassPro Shop)	$40.00
Gloves	Nitrile or latex gloves should be worn when handling rodents, tissue/blood samples, and traps to prevent transfer of pathogens and ticks.	Varies	Varies
White coveralls	Protect against tick bites and pathogens. White emphasizes dark color of tick and assists with location.	Cloth (washable): Red Kap Men’s Speed suit (AutomotiveWorkwear.com) Disposable: Uline Deluxe disposable coverall (Uline)	Cloth: $40.00 Disposable: $4.00/suit
Glasses or goggles	In some situations, vegetation may be thick and there is a risk of eye injury from twigs and branches. Eye protection can be considered if this risk is identified.	Varies	Varies
Duct tape, packing tape, or similar	Sticky tape wraps around ankles and the tops of boots to prevent tick access to legs through pants or socks.	Varies	Varies
Repellents	CDC approved repellent or pyrethroid impregnated clothing	See the EPA repellent search tool (EPA 2019)	Varies
Sun protection	Wide brimmed hat and/or sunscreen	N/A	Varies
Spider guard	Hat with arthropod mesh and a 1 m rod or stick	Sea to Summit hat and net (L.L. Bean)	$14.95
Hand sanitizer	Should be used in concert with nitrile gloves after handling anything mice have contacted	Varies	Varies

^1^Suggested equipment include protection from risks associated with terrain, vegetation, small mammals, and ticks. This list is not exhaustive and may need to be modified for individual circumstances or institutional requirements.

### What Type of Trap Should I Use?

Sherman live traps (H.B. Sherman Traps, Tallahassee, FL) are recommended and one of the most commonly used types of traps for rodent studies (Table 2). Sherman traps are a single-occupant rectangular prism made of solid metal, either aluminum or galvanized metal. Sherman traps have a treadle on the floor that when tripped, releases a door that closes the trap. Traps are available in many models including folding/nonfolding and perforated/nonperforated models of various size. Studies on the advantages or liabilities of small or large Sherman traps have been inconclusive. Some studies suggest smaller Sherman traps are more effective (Maly and Cranford 1985, Whittaker et al. 1998, Anthony et al. 2005), while others suggest larger traps (Slade et al. 2018) or that there is no difference (Kisiel 1972, Maly and Cranford 1985). Ultimately, the size and configuration of the Sherman trap used for any study will depend on preference, financial resources, and climate.

**Table 2.** Traps and trap maintenance and preparation supplies including examples and estimated cost

Item	Purpose	Example	Estimated cost
Sherman traps	Various sizes and styles are available	Shermantraps.com	$22.00–$32.00
Extra trap pins	Replacements for lost or bent pins	Shermantraps.com	~$1.00
Research labels or paint	Stickers or paint to mark Sherman traps as research equipment with the project leader information	Uprinting	~$0.50/sticker depending on options
Bleach or lysol	Cleaning materials for traps	Varies	Varies
Bucket or hose and sprayer	Hose with spray nozzle or 5-gal buckets to hold water for trap soaking	Varies	Varies
Scrub brush	Scrub traps of rodent waste and excess bait	Varies, but bottle brushes are helpful	Varies
Plastic zipper freezer bags	Storage of bait from unused traps	Varies	Varies
Biohazard bags	Disposal of waste materials from trap cleaning	Bel-Alert SP Scienceware biohazard disposal bags (Fisher Scientific SKU F1316414190)	$159.50/200 bags
Autoclave tape	Disposal of used materials from trap cleaning	Varies	Varies

Other traps (e.g., Longworth traps, Longworth Scientific Instrument Co., Oxford, England; Ugglan traps, Granhab, Gnosjö, Sweden, and others) have also been used frequently in rodent studies, but Sherman traps have been more successful than others in many habitat types and climates resulting in reduced mortality and increased captures (Sealander and James 1958, Anthony et al. 2005, Torre et al. 2010). However, there have been conditions where other traps have outperformed Sherman traps (Jung 2016); thus, it is important to select traps within the context of the study design, species assemblages, and the target ecological region.

### How Often Should I Trap and How Many Traps Should I Use?

*Peromyscus* spp. are nocturnal rodents so trapping is conducted overnight. Traps should be set as late in the afternoon as possible (around sunset) and opened as early as possible (sunrise). Previous research has suggested that interference with traps by larger nontarget species can be reduced by setting traps later in the day (Roden-Reynolds et al. 2018).

The number of nights in a trapping session and number of total trapping sessions will differ depending on study requirements. Increased trap numbers may reduce how many nights are needed but will require more intense labor daily to check and process efficiently. If the purpose of the study is to conduct tick surveillance related to *Peromyscus* spp. population size, multiple trap nights may be required which allow for recapture evaluation, mark-recapture calculations, and allow for a greater proportion of the local population to be trapped. This method increases effort during a single-trap session but may only need to be conducted once/month or season. If tracking tick abundances or pathogen infection over a season is desired, a lesser number of traps with more frequent trapping events (i.e., weekly or bi-weekly) may be necessary. Effort may need to be modified based on the number of recaptured individuals from one capture event to the next as well. Besides the standard ‘trap prone’ (those that get recaptured often) or ‘trap shy’ (those that will not be captured or recaptured) individuals (Gurnell 1980), the populations may be low and there may be frequent repeat captures. The ultimate number of traps set per night and number of consecutive nights is heavily dependent on financial resources and personnel availability. Careful consideration should be made in the study design and resource availability, so appropriate data are collected and animal welfare remains a priority. Early in the season, cooler temperatures will permit longer processing windows, but in areas where summer heat may be of concern to animal welfare, more personnel will be required to process higher numbers of captures before the heat of the day. Keep in mind that processing time includes not only physically handling mice but also retrieving traps and releasing captures which may require hiking to and from trap locations, depending on access. A species accumulation curve analysis may help with assessing required trapping effort (Colwell et al. 2004).

Total trapping effort is typically measured by number of ‘trap nights’, the cumulative number of traps deployed multiplied by the number of trapping nights in a trap session, then multiplied by the number of sessions. For example, if 10 traps were set on a transect line 3 d in a row each of 4 wk, the total number of trap nights would be 120 (10 traps × 3 nights × 4 wk). Falsely sprung traps make the trap unavailable for capture for a certain portion of the night which would impact total trap nights. These can be accounted for when calculating trap nights by applying the correction of (Nelson and Clark 1973).

### How Should Traps Be Arranged?

When setting live traps, it is important to establish a permanent trapping layout. Two spatial designs are primarily used to deploy traps in the field: grids and transect or line trapping. Grid trapping places traps on a grid with parallel and perpendicular lines. Line or transect trapping is conducted when traps are set in a single line. There is a lack of consensus on the ideal method for sampling small mammal communities and the best method depends on the goal of the study. In general, grids provide better spatial resolution for estimating population density, depicting home ranges, and determining small-mammal dispersion, which could inform hypotheses related to tick distribution. Transect or line trapping requires less effort per site because fewer traps are set generally and/or they are placed linearly requiring less distance traveled. This method better reflects community composition and provide better samples for examining demographic attributes such as age and sex ratios and habitat relationships due to greater numbers of captures, individuals captured, and species captured which may be beneficial to hypotheses related to individual host–tick interactions. Transects, however, suffer from an extensive ‘edge effect’ (Flowerdew 1976), where there may be a disproportionate number of mice captured than in the general area; thus, it is a relatively poor method for assessing densities or absolute abundance but entirely appropriate if comparing treatment effectiveness on tick burdens and pathogen infection between study sites. Transect arrangements may be more suitable when populations are low (Pearson and Ruggiero 2003).

### Where Should Traps Be Placed?

Transect and individual trap placement should be thought about carefully. There have been mixed reports of mouse density in edge habitat over forested habitat (Cummings and Vessey 1994, Bayne and Hobson 1998, Manson et al. 1999, Hoffmann et al. 2010), whereas others have reported no difference (Nupp and Swihart 1998, Anderson et al. 2006). Individual traps should be set in the most suitable habitat for the targeted species. For *Peromyscus* spp., those are areas with more plant cover (McCay 2000, Machtinger and Li 2019).

There are two considerations for trap placement: 1) site of the study and 2) microhabitat for individual traps. Study site selection may or may not be flexible depending on study goals and design. *Peromyscus* spp. are widespread and have a range of suitable habitats; however, there are likely to be site-specific differences. Therefore, it is important to characterize each study location including topography, elevation, and vegetation as well as tick abundance and/or density for site comparisons. For vegetation analysis, various methods have been developed depending on research needs and can be found in Bookhout (1994) and Silvy (2012).

Ideally, individual trap placement should flat to the ground and in-line with a runway such as a log or tree that can act like a drift fence, or near a hole or hiding place in a tree. In areas without such structure, placing traps within shrubby areas will reduce glare from the trap metal and be more inviting to mice. New traps are very reflective, so often adding leaves or grasses to the top and/or sides of the trap can reduce glare as well and promote capture success.

### How Will I Find My Traps?

Prior to the first trapping session, sites should be evaluated, and markers placed. A meter wheel can be used to determine correct distances between traps, but this tool may be difficult to use in rocky terrain or areas with woody debris so a measuring tape or human pacing can be used. Keep in mind that if trapping starts early in the spring, vegetation may change throughout the season and may need to be cut back enough to allow for personnel to properly travel and place and recover traps. Keep vegetation maintenance as minimal as possible to maintain habitat for mice.

Trap locations should be marked with utility stick or wire flagging (ground) and/or flagging tape (tree) for quick identification (Table 3). Flagging colors should not interfere with already present flagging (such as gas or water line) and should not be green or yellow as these are difficult to find when vegetation is thick or during the fall when leaves change color. Neon orange and pink are typically preferred. Flagging should be visible from trap location to trap location or be used to guide trappers to each station. Not only does this increase speed of trap deployment, recovery, and rebating but prevents against trap loss. Trap locations should be identified by a unique identifier on the flag and tape and marked consecutively as another check to ensure traps are not missed. This is particularly important if traps are removed to a central location for animal processing and need to be returned and released. Flagging should be minimized if trapping in residential backyards due to aesthetic concerns of cooperating homeowners and all markers should be removed at the conclusion of the study. Finally, GPS coordinates should be taken of each trap location for future reference and mapping.

**Table 3.** Field location marking and preparation supplies including examples and estimated cost

Item	Purpose	Example	Estimated cost
Utility flag stakes	Mark trap locations	Empire pink flagging stakes (Home Depot SKU 1,002,378,475)	$7.98
Flagging tape	Mark trap location and facilitate easy travel from one point to the next	Empire pink flagging tape, (Home Depot SKU 114348)	$5.97
Reflective markers	May be useful in dense vegetation and can assist with trap line trail location	Presco stripe vinyl flagging (Forestry Suppliers SKU 57988)	$2.65
Permanent marker	Marking flags and tape	Varies	Varies
Handheld GPS device or GPS App	Documenting GPS locations of traps for future mapping	Appliance: Garmin Oregon 700 App: HandyGPS	Appliance: $289.99 App: $6.99
Vegetation lopper	Removal of dense vegetation for easier travel	Fiskars Bypass Lopper, (Home Depot SKU 91416966J)	$34.98
Meter wheel	Marking trap line distances	Komelon 60 Series 19” metric wheel (Komelon)	$49.00

### Should I Protect My Traps From Other Animals?

**Fig. 2.**
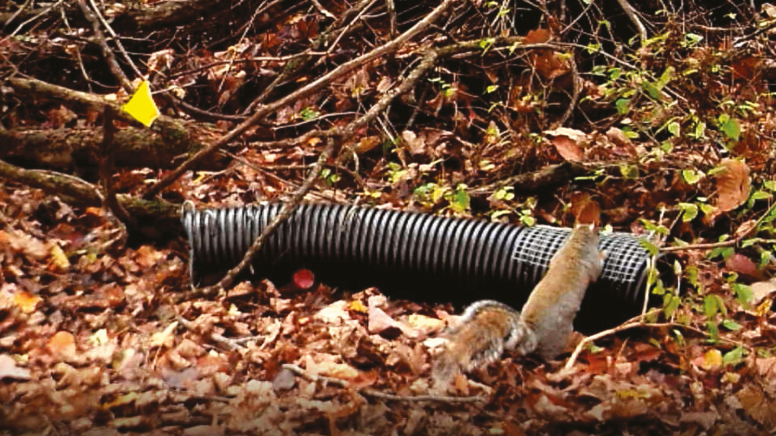
Protection of Sherman traps can be added if there is excessive non-target animal interference. Photo courtesy E. T. Machtinger

In some areas, Sherman traps can have a high rate of failure from predators and other wildlife (Layne 1987, Atkinson 2009, Getz and Batzli 1974). Raccoons (*Procyon lotor* L. [Carnivora: Procyonidae]) and eastern gray squirrels (*Sciurus carolinensis* Gmelin [Rodentia: Sciuridae]) can quickly locate baited traps and trigger or move them, or even predate on trap captures. These species also may become conditioned to trap placement and can become repeat offenders. While frustrating, methods have been developed and tested with success in reducing nontarget influence on Sherman live traps during tick surveillance. Trap lines can be moved (Barnett and Dutton 1995) or nontarget exclusion devices can be applied to Sherman traps (Fig. 2; Roden-Reynolds et al. 2018). Preliminary trapping can give an idea of the extent of interference so that the study is not affected (see *What If My Capture Rate Is Low?* section).

### What Bait Should I Use?

Baiting is a complex topic and preferences for type, amount, and duration of baiting can differ among researchers. Prebaiting, or baiting traps in place but keeping them secured open for one or more days prior to a trapping session has been suggested to reduce trap avoidance. However, Edalgo and Anderson (2004) found that trap success was not increased by prebaiting. Prebaiting may only be beneficial if trap sessions are one to two nights (Gurnell 1980).

**Fig. 3.**
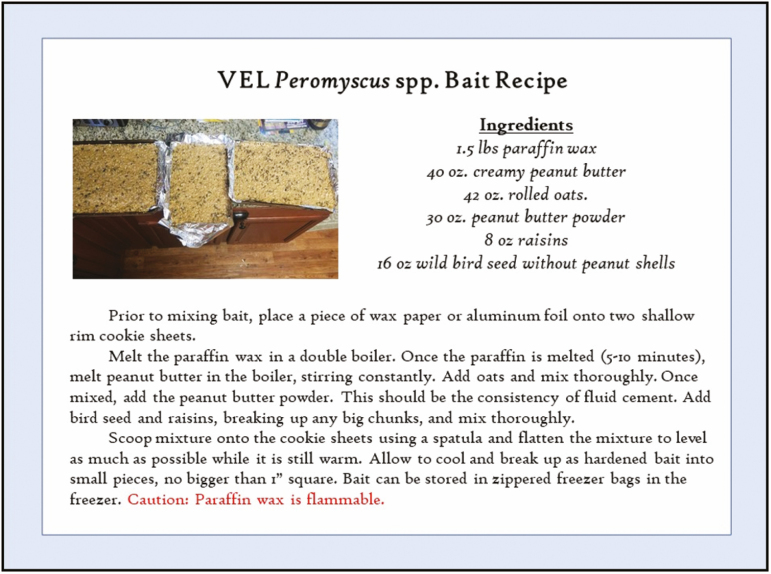
VEL bait recipe for *Peromyscus* capture.

Because foraging is typically odor based in *Peromyscus* spp., the North American standard for bait is some combination of peanut butter and oats (Schemnitz et al. 2009), although even peanut butter cracker sandwiches have been used. In other cases, handfuls of birdseed, vanilla extract, or other materials have made effective trap bait; ≥1 oz (28 g) is typically required per trap night. A recipe that has worked well for projects in several regions is provided in Fig. 3. Whatever bait is selected, it should be standardized and used throughout the project to prevent capture bias unless mitigating for low captures or other trapping problems.

Along with an odor bait, generally two to four medium cotton balls should be provided in each trap for warmth and to provide nesting material for trapped rodents as well as a succulent such as a 8- to 16-g piece of potato, carrot, or apple for moisture (approximately 1/16 to 1/8 of an apple). Regardless of bait used, it should be placed in the back of the trap with the cotton to encourage the target to enter the trap and to not interfere with the treadle mechanism.

If multiple nights of trapping are anticipated, rebaiting of traps is made easier if each trapper has a bag of succulent, cotton, and bait. Traps can easily be rebaited as needed and opened by walking the trap line or grid instead of picking traps up during the day. In some locations, arthropods may also be attracted to bait, specifically ants. If ants are a problem, bait can be made by soaking cotton in a peanut butter and water mixture as described by Atkinson (2009) and placed in the trap along with the cotton nesting material. Baiting supplies can be found in Table 4.

**Table 4.** *Peromyscus* spp. bait supplies including examples and estimated cost

Item	Purpose	Example	Estimated cost
Bait	To attract rodents	See Fig. 1	~$0.22/oz with peanut butter powder ~$0.17/oz with only peanut butter Reusable
Succulent piece	8–16 g of apple or similar fruit or carrot or potato to provide moisture for trapped rodents	Varies	Varies, ~$0.08-$0.16/trap
Cotton balls (medium)	Nesting material for trapped rodents	Varies	Varies, ~$0.02-$0.04/trap, reusable during a trapping session
Plastic zippered freezer bags	Storage of bait	Varies	Varies

### What Preparations and Maintenance of the Traps Do I Need to Make?

**Fig. 4.**
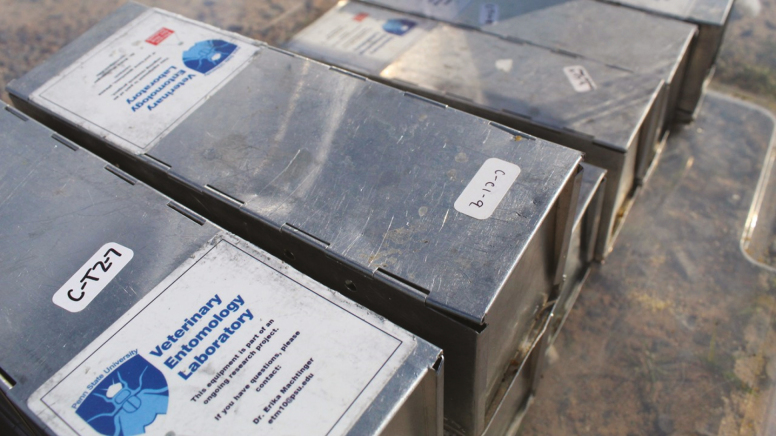
Sherman traps should be identified with paint or a sticker that includes at minimum the contact information of the project leader. Smaller stickers can be used to temporarily identify Sherman traps recovered from the field with a captured animal to facilitate returning that animal to the appropriate location. Photo courtesy Taylor Miller.

There are a few preparations that must be made to Sherman traps prior to setting in the field: 1) unfolding traps if a folding model was purchased, 2) setting the trigger mechanism sensitivity, and 3) labeling traps (Table 5). The effectiveness of Sherman traps relies on the sensitivity of the trigger mechanism; therefore, it is essential to check the release mechanism prior to setting traps. A light finger press should trigger the mechanism. If this is not the case, pressing the tab that holds the trigger either back or forward can change the sensitivity. Caution should be taken to make sure the trap is not so sensitive that movement of the trap will trigger it as this will reduce overall captures. Another optional preparation is adding labels or metal paint to traps for research identification. The wording on these labels is study specific, but at a minimum should include the name and contact information of the project leader (Fig. 4; Table 2).

**Table 5.** Trap deployment and recovery supplies including examples and estimated cost

Item	Purpose	Example	Estimated Cost
Stickers	Placed on traps so that trap capture locations can be marked for easy return for capture release.	Removable adhesive industrial thermal transfer labels (Uline SKU S-9631)	$27.00
Permanent markers	Mark trap stickers with location of trap capture for easy return for capture release.	Varies	Varies
Trap transportation	Laundry bags or similar sacks can be used to deploy large numbers (>20) traps in the field at a time. Plastic bins or milk crates can be used to transport traps to return trapped rodents to a central processing location.	Varies	Varies

Maintenance of Sherman traps is generally minimal. There are a few studies that have evaluated attraction of *Peromyscus* spp. to traps with conspecific odors (Mazdzer et al. 1976, Drickamer 1984, Wolf and Batzli 2002), which may increase trap captures. After a trapping session (which may be one or more consecutive days), hinge pins can be removed so that traps can be completely opened and bait and cotton removed. This can be labor intensive, thus if trap cleaning is effective without removing the hinge pin this may save time. A trash bag should be available when cleaning out cotton and other debris from traps. In areas where hantavirus is a concern or if your institution requires it, this should be a biohazard bag. Traps can be cleaned with a scrub brush and a 5–10% hypochlorite bleach or other disinfecting solution rinsed in a bucket of clean water or hosed, and dried. This should be conducted outside if possible, especially in areas at risk for hantavirus infection. Disinfectant use does not appear to influence trap captures (Van Horn and Douglass 2000, Kaufman et al. 2011, Wilson and Mabry 2011).

Depending on bait type, baiting can occur prior to trap deployment or during trap placement. Premade baits, cotton, and succulents can be added prior to deployment as these are often too large to get stuck under the treadle, but loose seeds or oats may move around too much during transport and interfere with trap function. Baiting traps can occur a day in advance if temperature allows (i.e., peanut butter will not melt) which can help with the efficiency of trap deployment. Carrying traps is easily done with either a plastic bin or crate (a legal crate holds a dozen traps), or a large tote bag or laundry bag that can be carried over the shoulder (Table 5).

### What If My Capture Rate Is Low?

Capture success typically varies due to area, season, or trapping effort and in our experience can range from 0 to 50% or even more. If capture success is lower than expected, mitigation measures can be employed to increase the number of trapped individuals (Table 6). To avoid impacting the study, it is ideal to have a preliminary trapping session to determine ideal trap locations and estimate *Peromyscus* spp. population density, as well as determine if there will be any animal interference with traps. A preliminary trapping session in the year of the study can provide important feedback on potential trap numbers, animal interference, recapture rates, and other concerns but could also impact trap happy and trap shy behavior. In northern climates trapping in the early spring may not provide much feedback as populations are likely to be low, so preliminary sessions should be considered in the fall or summer prior to initiating the study.

**Table 6.** Possible reasons of low trap capture success of *Peromyscus* spp. and suggested mitigation measures

Reasons for low-trap captures	Suggested mitigation methods
Low density of target species	Assess if the experiment can be relocated to a different plot or more suitable habitat. Home ranges of some *Peromyscus* spp. overlap, so increasing trap numbers may increase trap captures, but this is not always the case. Marking animals during the first capture round and calculating the recapture rate can help provide an estimate of density (see *What If My Capture Rate Is Low?* section).
Weather interference	Weather and moon phases should be taken into consideration. Small mammals adjust their activity based to reduce predation risk. Thus, *Peromyscus* spp. trap captures will generally be higher when moon illumination is low and during cloudy or damp nights (Orrock et al. 2004, Fanson 2010).
Treadle failure	The tripping mechanism on the treadle should be checked and adjusted prior to each trapping round (see *What Preparations and Maintenance of the Traps Do I Need to Make?* section).
Trap interference by other animals	Trap interference from non-target captures can be mitigated by deploying different trap types or baits concurrently or relocating traps. Manipulation of traps by mesocarnivores or other animals can be reduced in some situations by trap relocation, but also with protective exclusion devices (Roden-Reynolds et al. 2018; 2.1.7)
Obstructed entrance	Traps placed too close to a structure, woody debris, or vegetation to allow for easy entrance should be repositioned.
Insufficient bait	*Peromyscus* spp. are attracted to many types of baits. However, if a bait with limited odors is being used (i.e., just seeds) and trap capture numbers are lower than expected, provide peanut or peanut butter-based bait to increase range of odor attraction (see *What Bait Should I Use?* section).
Inappropriate location of trap	Traps should be placed in areas where *Peromyscus* spp. are most likely to travel and forage (see *Where Should Traps Be Placed?* section).
Trap too reflective or too much light	Try to place trap where light will not reflect of surface of traps. If traps are new, using leaf litter or grass to cover some or all of the reflective surface may be beneficial.
Trap not functioning correctly	Traps should be cleaned and inspected after each trapping session. However, if a trap is suspected to be failing in the field, the trap should be replaced and the removed trap inspected and repaired, if possible (see *What Preparations and Maintenance of the Traps Do I Need to Make?* section).

### How Can I Protect Against Unintentional Mortality of Captures?

Unintentional mortality of target and nontarget captures are not always preventable, but animal welfare is a priority in all circumstances so every effort must be made to avoid situations where mortality risk may be increased. Rates and an analysis of causality of mortalities are reviewed in Lemckert et al. (2006) but common causes of mortality include damp bedding, lack of appropriate food, excessive heat or cold, exposure to the elements, or carnivore interference. Some of these risks can be planned for and avoided, whereas others may be site-specific and may need to be addressed in the course of the study. Suggestions for mitigating these concerns are highlighted in Table 7.

**Table 7.** Mitigation suggestions for mortality risks associated with *Peromyscus* spp. Trapping

Risk	Suggested mitigation methods
Damp bedding	● Provide ample cotton (see *What Bait Should I Use?* section). ● Reduce or eliminate trapping during heavy rain events. ● Cover Sherman traps with a wooden or metal ‘weather shield’, or a 2-liter milk carton cut at either end so the trap slides in (or similar) to prevent additional moisture in the trap. ● Do not place traps at the bottom of hills or other areas that may accumulate water or runoff.
Insufficient food	● Provide enough bait to last the duration of the trapping period. ● Provide moisture with a slice of apple, carrot, or potato (see section *What Bait Should I Use*?).
Temperature extremes	● Reduce or eliminate trapping during extreme heat, or open traps earlier in the morning. ● Reduce or eliminate trapping when temperatures are under 4°C if the research permits.
Anesthesia risks	● Understand how temperature affects efficacy of Isoflurane. ● Understand how age, sex, and reproductive status may influence Isoflurane efficacy. ● Be familiar with signs of respiratory distress including irregular breathing patterns (gasping). ● If irregular breathing patterns emerge, immediate remove mice from the anesthetic jar, stimulating the body with gentle manipulation for recovery and relocate to home trap with a hand warmer to increase body temperature.
Carnivore interference	● Protect traps with exclusion devices (Roden-Reynolds et al. 2018).
Species specific risks	● Shrews are at high risk for mortality due to a high metabolism. They should be immediately released at the site of capture.

### Handling Captured Animals

It is strongly recommended for all medical procedures (anesthesia, euthanasia, tissue samples), if not required by the IACUC, that the researcher have training with an experienced researcher or veterinarian to learn proper handling techniques. If possible, methods can be practiced on laboratory mice before performing these functions in the field and to learn the potential risks and unintended results that may require mitigation (i.e., respiration depression, hemorrhage during blood draws, etc.). Many institutions will have training laboratory colonies of mice, so even if your institution does not, you could reach out to a local University animal resource program.

**Fig. 5.**
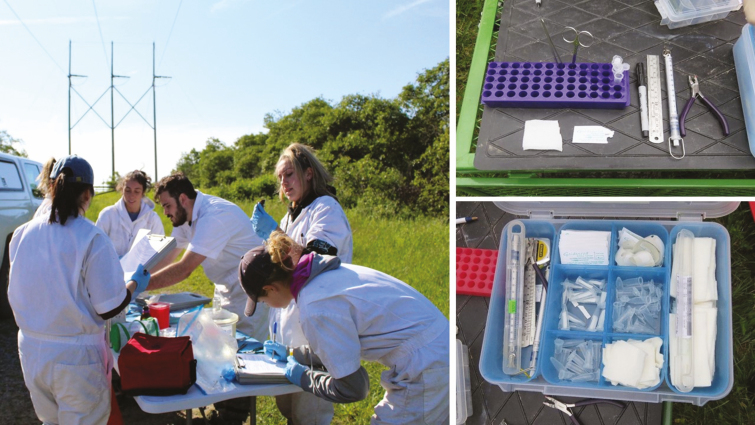
A central processing station should be set up near the trapping lines to reduce animal stress and expedite processing (left). Before animals are processed, all materials and supplies should be organized and prepared (top right). A multi-divider kit can be helpful to organize supplies (bottom right). Photos courtesy Taylor Miller.

Before leaving for trap checking and capture processing, all equipment should be cleaned and double-checked. Trap supplies can be organized and prepared in advance including cryolabels on vials, filter paper strips cut to fit in vials, ethanol, and/or RNALater (Ambion Inc., Austin, TX) in vials, etc. Most equipment can be stored in an organization bin or toolbox (Fig. 5), which can help with set up efficiency. In addition, vial holders (Fig. 5) can be very helpful to organize vials (blood, tissue, and ticks) in a specific order for processing. All trapping supplies can be stored and transported in a suitable plastic bin with a locking lid. These trapping kits are useful not only to organize supplies, but if there are multiple trapping stations a kit can be assigned to each station.

Animal welfare is of course a primary concern, and as such, captures should not be individually processed at the site of capture, but instead transported to a central processing station (Fig. 5). This designated area should be immediately adjacent to or within a trapping grid where all processing equipment will be laid out.

### Examination of Traps and Recovering From the Field

**Fig. 6.**
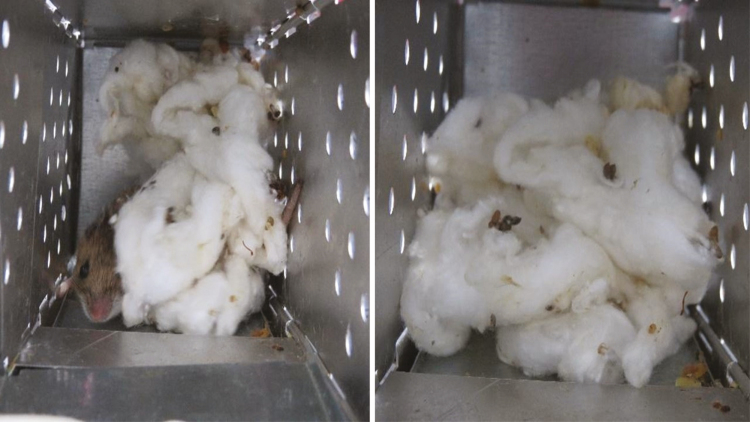
*Peromyscus* spp. will typically ‘fluff up’ provided cotton and this is a fairly good indicator of a successful capture. Photos courtesy E. T. Machtinger.

Identifying trap locations should be relatively simple if traps have been marked (see *How Will I Find My Traps?* section). Collection bins or crates can be used to aid in trap pickup and transport. Placing traps in crates in the same order they were gathered ensures accurate recording of which traps were occupied and which were not. A good practice is to place unoccupied traps face down with the door opened and occupied traps face up (sticker or paint side up) with the marked front door closed. If you have multiple trap nights, unoccupied traps can be left in the field, but the trap doors must be shut until the next trap evening. A trap with its door still open will be unoccupied, however, not all sprung traps will contain animals. Wind, rain, falling objects, and other animals will trigger traps. When approaching a closed trap, listen for an animal within. If you do not hear anything, pick the trap up and evaluate weight and odor. Rodents have a musk that will be distinctive after some experience. If occupancy is not confirmed with these indicators, gently open the door slightly to visually inspect inside. Keep in mind that small animals can and do hide underneath the treadle so be sure to inspect there as well. Rodents will typically ‘fluff up’ the cotton (Fig. 6) provided, and even if you do not immediately see an occupant, the trap weight and ‘fluffed’ cotton is often a good indicator.

If the trap is occupied with a target species, place it in your carrying crate and move on to the next. If there is a nontarget, release it at the trap site. Because trap captures should be released back at the site of trapping, it can be useful to carry a marker and blank stickers (Fig. 4). When a capture is identified, place a sticker on that trap and mark the trap number. In this manner, the trap locations can be identified even if they are moved out of order (Table 5). Take collected traps to the central processing location. When gathering traps, be sure to return with the same number that were deployed the previous day. While nontarget animals do relocate traps on occasion, leaving a potentially occupied trap in the field for an animal to suffer or perish due to human apathy and/or incompetence is not acceptable.

### Removal of Animal

Once all traps have been gathered and you have returned to the central processing location, be sure that animals in traps are not stressed and are comfortable. If they appear cold, place occupied traps in direct sunlight or on the hood or warm engine block of the parked vehicle until warm. HotHands heaters (Kobayashi Consumer Products, LLC, Dalton, GA) can be used under the trap or inside the trap wrapped in a thin towel to increase trap temperature, just remember to activate the warmers prior to collecting traps. If captured animals appear too hot, place them in the shade. There is some degree of trap stress, but thermally stressed animals cannot handle anesthesia well and mortality rate will increase without precautionary measures.

**Fig. 7.**
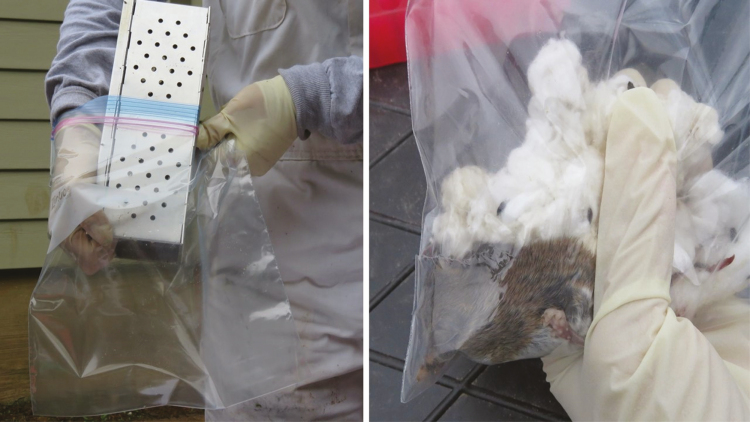
If trap captures need to be removed prior to anesthesia, the Sherman trap can be held upside down in a plastic bag. The trapper gently presses down on the trap door with the hand that is in the bag (being careful that the capture isn’t under the door) and holds the bag tightly with the other hand around the trap and the arm to prevent escape (left). Captures can be manipulated into the corner of the bag so they can be observed and/or scruffed (right). Photos courtesy E. T. Machtinger.

Animals typically are not willing to come out of traps, so will need some encouragement. Place the entrance of the trap in a 1 gal or larger plastic bag and tightly hold the top of the bag around the trap with one hand. Initially, place the bag with the door entrance facing up. Otherwise, opening the door may actually trap or crush the mouse if it is located near the entrance. The door of the trap can be opened with the fingers and then oriented downward. The person’s hand should be folded over the bottom of the trap so that the fingers can open the trap inward (i.e., trap should be ‘upside down’; Fig. 7). This allows the animal to slide down the roof, where there are fewer internal components to cling to. The person holding the trap should use their fingers to keep the trap open and gently shake occupants into the plastic bag. Slide the trap out of the bag while keeping the bag tight around the trap and then closed so that the animal cannot escape. To remove bedding and other material, orient the animal so that its head is in a corner of the bag and then circle the animal from outside to prevent movement (Fig. 7). Using your other hand, reach into the bag and scruff the animal firmly. Individual animals differ in degrees of vocalness or activity while being handled. If an individual capture struggles, it can be held gently with the feet on the handler’s chest and/or the tail can be supported while being held by the scruff of the neck. With the bag method, the animal can be weighed, identified to species (primarily nontarget species), and ear tags observed prior to anesthesia and reactions to anesthesia and timing can be observed. However, transfer of trap captures may result in escapees, especially with less experienced handlers. If species identification or individual identification is not required prior to anesthesia, or if handlers are not comfortable handling mice when they are not anesthetized, mice can be transferred directly to a jar or a bag described above containing an anesthetic.

### Anesthesia

Pain and distress may be unavoidable during some handling procedures, such as sampling tissue or blood, and the IACUC will likely require use of an anesthetic in order to perform such tasks. In addition, removing small ticks may require immobile individuals as larvae and nymphs are difficult to sample and often are located near sensitive areas like the eyes.

There are only a few anesthetic options for use on small mammals. Subcutaneous or intramuscular injections of Ketamine or Ketamine/Xylazine in combination result in a long working window (upwards of 45 min) to perform procedures. But there are significant draw backs including the fact that Ketamine is a Schedule III controlled substance which requires a federal permit to purchase and possess. Additionally, animals may not be fully alert when researchers are ready to release them, making them more prone to predation. Details on this method can be found in Tsao et al. (2004) and Peavey and Lane (2017).

The inhalant anesthetic Isoflurane is the most widely used as it is the easiest and least expensive (Table 8). Isoflurane has a very fast induction time, resulting in a small window when animals are at the surgical plane of anesthesia in which to perform any painful or distressing procedures. The open drop method is the easiest for field use in which a small amount of Isoflurane is placed on a cotton ball with an eye dropper or plastic pipette and placed in a 1-gal, zippered plastic bag or glass jar. Which option to use will often depend on space, needs, and IACUC requirements (some may not permit mouse contact with anesthetic during induction which can be mitigated by using a desiccator jar with a porcelain plate with holes to separate mice from treated cotton, or a tea strainer to hold the cotton). Mice can be placed in the plastic bag directly from traps as described above. In addition, Isoflurane does not seem to effect on-host parasites like ticks and fleas.

**Table 8.** *Peromyscus* spp. removal and anesthesia supplies including examples and estimated cost

Item	Purpose	Example	Estimated Cost
Temporary heat	Warm chilled mice and assist with temperature regulation during anesthesia recovery	HotHands Hand Warmers (Uline SKU S-14297B)	$38/40 packets
Plastic bag	A plastic bag can be used as an anesthetization chamber, holding chamber, or observation chamber	Hefty Jumbo storage bags (2.5 gal)	$13.41/45 bags
Isoflurane	Anesthetic	*Must be purchased by a licensed veterinarian*	$72.95
Cotton balls	Material that anesthetic is placed on	Varies	Varies
Glass jar (alternative to plastic bag/optional)	Anesthesia chamber	PYREX Knob top nonvacuum glass desiccators (Fisher Scientific SKU 08-624-411)	$341.21
Tea strainer (optional)	Provides separation between animals and anesthetic	Varies	Varies

Bags can be closed around mice and their status monitored closely such that they are quickly removed when respiratory rate reaches about one breath/second. Because Isoflurane is a respiratory depressant, animals left exposed longer will likely succumb to effects and die. If mice become alert during handling, they can be placed back into the bag until the appropriate respiratory rate is reached again. It is important to recognize that Isoflurane is highly volatile and should only be used outdoors with caution and replenished as necessary. The effect on captured rodents will vary with temperature and by individual, so time to surgical plane of anesthesia is not standard and should be monitored closely. Isoflurane also interferes with rodent temperature regulation during recovery, so HotHands heaters can be used (remember to initiate heating before traps are picked up) to aid in recovery. More information on this method can be found in (Mills et al. 1995).

### Euthanasia

Euthanasia of mice may be part of your research protocol or may be reserved as a humane alternative to releasing an obviously distressed or injured animal. While there are several acceptable humane euthanasia techniques, the easiest and least distressing to animal subjects and researchers alike is anesthetic overdose with 5-ml Isoflurane (Parker et al. 2008). Subjects to be euthanized can be placed in the bag with a proper dose of Isoflurane until movement ceases. If animals are moving in the bag, be sure to refresh Isoflurane and wait until movement ceases. Once respiratory and extremity movement ceases, pinch the toe or tail to detect any reactions. In most cases, 5 min should be more than enough time. When the animal has died, a secondary form of euthanasia is typically performed. This can be exsanguination via cardiac puncture or cervical dislocation. Acceptable euthanasia techniques are described in Leary et al. (2020).

### Species Identification

In 1909, the genus *Peromyscus* was revised and recognized that while superficially similar, *P. leucopus* and *P. maniculatus* were different species (Osgood 1909). Since then, phenotypic characteristics like dental morphology, cranial size, tail length, and degree of bicoloration of tail and/or pelage have been used (Waters 1963, Choate 1973). The American Society of Mammalogists recommends using standardized measurements of total length (body + tail), left hind foot, and ear length. Feldhamer et al. (1983) used the tail length to head and body length to weight ratios to distinguish between these two species with >93% accuracy. Similarly, discriminant function analysis of tail and body length, ear size, and weight was 92% accurate at identifying species. However, *Peromyscus* spp. are known to have morphological variations based on location (Kamler et al. 1998, Grieco and Rizk 2010); thus, these measurements have performed poorly in the field in other circumstances (Bruseo et al. 1999). In areas where these species overlap, it has been suggested that only molecular or biochemical methods are truly reliable at distinguishing species and that to avoid confusion or conclusions regarding tick ecology with questionable species identification, the species be referred to together as *Peromyscus* spp. in the literature unless additional diagnostic analyses have been conducted (Rich et al. 1996, Lindquist et al. 2003).

### Blood and Tissue Samples

There are several different permitted bloodletting techniques, all of which should be performed on anesthetized mice. The retro-orbital sinus bleed uses a capillary tube to rupture blood vessels behind the eye to obtain a blood sample. Its use is falling out of favor due to its unappealing nature and the fact that it can lead to ocular abnormalities in subject mice (Fried et al. 2015), which can negatively influence survival in the wild. The tail clip excises tissue at the tip of the tail and a small amount of blood can be sampled with a capillary tube (Golde et al. 2005).

An ethical and relatively easy to learn technique is the cardiac puncture (Diehl et al. 2001; Table 9). This technique uses a needle (25- to 27 gauge, 16 mm) and syringe (1 cc) to draw a blood sample (50–150 µl) directly from the sedated mouse’s heart. The wound clots immediately and mortality as a result of the procedure is very low. Mice need no postoperative care and can be released to the wild unharmed. However, as this method is also associated with euthanasia via exsanguination while under sedation, institutions may require justification for use if other options are available. It should be noted that death by exsanguination is not a requirement of the cardiac puncture technique and smaller blood volumes can be easily obtained without harm to the animal.

**Table 9.** *Peromyscus* spp. blood and tissue collection supplies including examples and estimated cost

Item	Purpose	Example	Estimated cost
Cardiac puncture: 1cc syringe/27 gauge-16mm needle combination	Blood collection	BD IV Insulin Syringe Fisher Scientific SKU 14-829-1D	$46.75/100 syringes
Submandibular puncture: Golden lancet	Blood collection	Braintree Scientific Goldenrod animal lancet (4-5mm) (Fisher Scientific SKU NC9416572)	$68.44/1000 lancets
Capillary tubes or filter paper	Blood collection	Varies	Varies
Microcentrifuge tubes or cryovials	Storage of samples	Fisherbrand Microcentrifuge tubes (Fisher Scientific SKU 05-408-137)	$63.70/500 vials
Vial storage box	Organization and long-term storage of samples in vials	Fisher Scientific 03-395-465	$12.36
Vial rack	Allows easy access to sample tubes that can be placed in specific orders facilitating quick processing	Cryogentic vial workstation rack, (Grainger)	$24.36
Cryovial labels	For identification of samples and long-term freezer storage	Fisherbrand Cryogenic labels for cryogenic storage, (Fisher Scientific SKU 15–910-D)	$52.00/1000 labels
Gauze	Stop excessive blood flow from rodents after blood draw	Varies	Varies
Ear punch	Removing standard size ear tissue for pathogen analysis	Fisherbrand animal ear punch (Fisher Scientific SKU 13-820-064)	$79.80
Ear tissue preservative	Preserve ear tissue for pathogen analysis	Invitrogen RNA*later* Stabilizing solution (Fisher Scientific SKU AM7021)	$454.00/500 ml
Ethanol/Flame	For sterilizing ear punch between rodents	Varies	Varies
Superfine forceps	For removing punched ear tissue from ear punch	Superfine Forceps (Bioquip Products SKU 4524)	$23.27
Sharps container	Disposal of needles and syringes and lancets	1-Pint Sharps container with lid (Hopkins Medical Products SKU 668901)	$3.75
Biohazard bags	Disposal of hazardous waste including used cotton and bait, gloves, and gauze	Bel-Alert SP Scienceware^-^ biohazard disposal bags, (Fisher Scientific SKU F1316414190)	$159.50/200 bags
Biohazard or similar cooler	Transportation of samples	Premium Insulated Bio Transport Cooler (Hopkins Medical Products SKU 530380)	$9.50
Cool or freezer pack	Transportation of samples	Varies	Varies

**Fig. 8.**
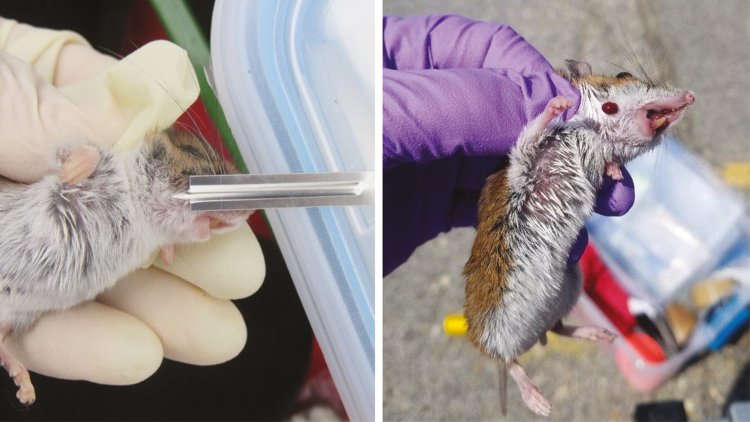
Submandibular puncture is a relatively simple process for blood collection, but it does require training and practice to master, especially on *Peromyscus* spp. Photos courtesy E. T. Machtinger.

Two additional methods of collecting blood that are relatively easy are saphenous vein collections or submandibular punctures. Saphenous vein draw may require additional mouse restraint, hair removal, and practice drawing blood from very small veins with a needle or lancet (Diehl et al. 2001). Submandibular punctures are a simple technique that uses a lancet to puncture the submandibular vein (Golde et al. 2005; Fig. 8). If using this method with *Peromyscus* spp., it is important that there is a good scruff on the animal so that the eyes are bulging and the skin is pulled tight across the skull. In laboratory mice, it is easy to locate the submandibular puncture site due to a prominent gland on the head. However, the head shape and pelage of *Peromyscus* spp. are different so this can often require some practice not only at proper location, but the appropriate angle of entry and force. There is nearly zero mortality as a result of this method, but because of locating the small vein, there can be times when a blood draw is not successful. Typically, two puncture attempts can be made for each side of the head before the draw is considered unsuccessful. A well-performed puncture will result in a drop of blood that is suitable for analysis (Fig. 8). If a larger than intended puncture is made or the animal continues to bleed, a pressure on a piece of gauze can be used to stop the bleeding. Depending on method, blood can be transferred to a microcentrifuge tube with a locking cap from the syringe, collected with a capillary tube, or absorbed on to a filter paper and transferred to a microcentrifuge tube. Blood samples should be placed in an upright rack in a cooler on ice. Upon returning to the laboratory, samples are typically centrifuged and sera removed from whole blood with a micro pipetter and stored separately between −20 and −80°C, depending on protocols.

**Fig. 9.**
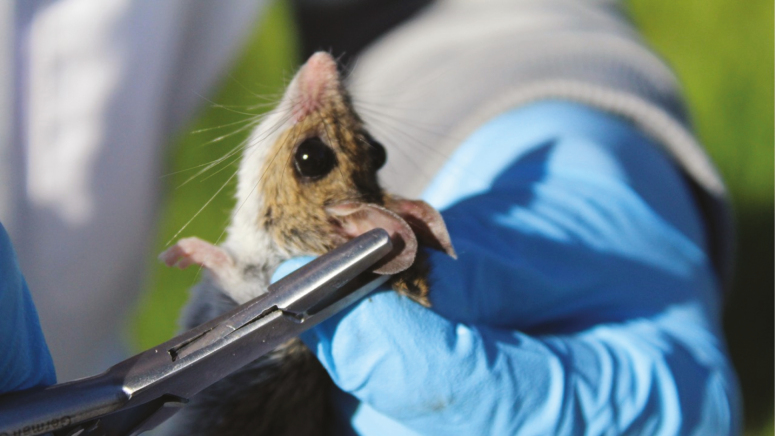
Ear punches/pliers are a standard way to take ear tissue samples in *Peromyscus* spp. that can be later tested for *Borrelia* spp. infection. Photo courtesy Taylor Miller.

Ear tissue should be sampled using a stainless-steel ear punch plier (Fig. 9). The plier-style is preferred over the scissors-style as the finger and thumb loops of the scissors-style can be cumbersome and fumbling with them wastes time during an already short working window. The 2-mm diameter works well for size consistency between samples and should be biopsied at the periphery of the ear when animals are sedated. The ear punch plier must be sterilized between uses using ethanol or a flame. Samples can be stored in locking-cap 2-ml microcentrifuge tubes with ~100 µl of an RNA preservative. Samples should be refrigerated overnight to allow preservative to fully penetrate and then samples can be frozen indefinitely. Be sure to check with those that will process samples to see what preservative they prefer and how they would like the samples handled.

**Fig. 10.**
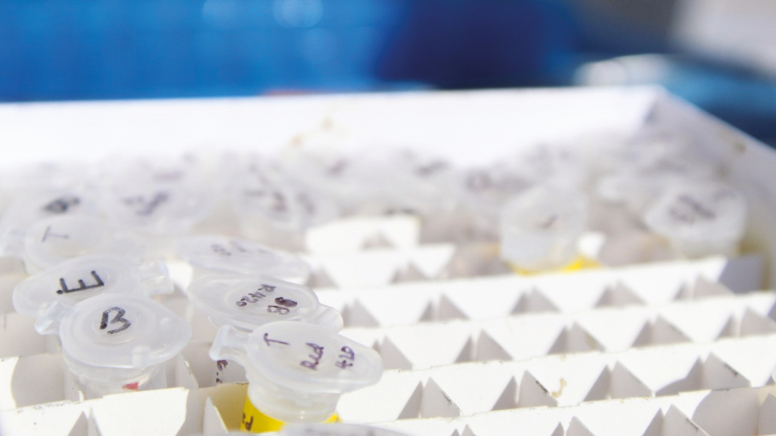
Coding tubes can help with organization and sorting after sample collection, especially if multiple types of samples are being collected (blood, tissue, parasites). Colored cryovial labels and/or different colored tubing is also effective. Photo courtesy Taylor Miller.

After blood and tissue collection, used needles and syringes must be placed in approved sharps containers and all waste should be disposed of in biohazard bags and autoclaved upon return from the field; never re-shield needles to avoid accidental needle sticks. Samples should be labeled and it can be helpful to have either different colored tubes or labels for samples or to use a coding system (i.e., B = blood, T = ticks, E = ear, etc.; Fig. 10) on the top of the tube so that samples can be easily sorted and organized.

### Weighing and Measuring

**Fig. 11.**
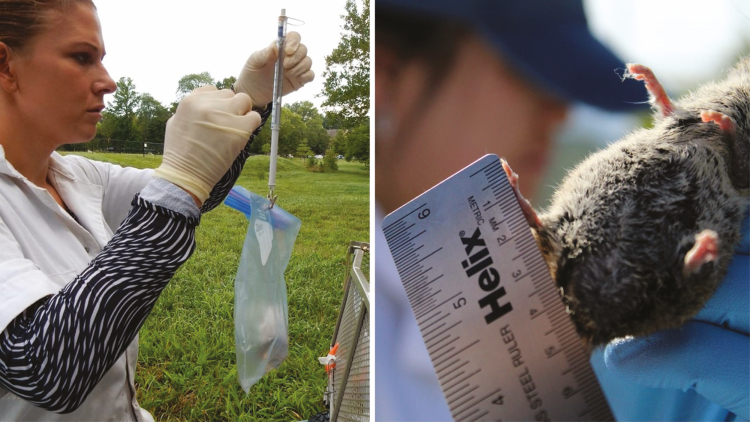
A spring scale (left) is used to weigh trap captures either while in a plastic bag or connected directly to the tail (with tape modifications to scale clasp). A metal ruler with mm markings is effective for taking morphometrics (right). Photos courtesy E. T. Machtinger.

Basic analysis of mouse size, sex, and age can aid in future ecological comparisons or assessment of demographic structure of trapped hosts among research sites or over time. Captured mice should be weighed and measured at each trapping event. While this process is not painful, it reduces stress if mice remain sedated or at least partially sedated. As a result, this is best performed as the last step of processing, just before mice are returned to traps. A vertical 30-g micro-line spring scale (Fig. 11; Table 10) will suffice for the majority of captures, though pregnant females and large males can and do exceed 30 g. In which case, a reserve 50- or 100-g scale can be used. The 30-g scale is preferred for accurate readings for the majority of mice. Tape can be placed over the teeth of the gripping mechanism of the scale which can then be attached directly to the tail of sedated mice. Or mice can be weighed in the processing bag and net mass determined by subtracting the mass of the empty bag. A small ruler with millimeter graduations can be used to measure body, ear, and hind foot lengths, if morphometric measurements are desired (Fig. 11).

**Table 10.** *Peromyscus* spp. weight and measurement supplies including examples and estimated cost

Item	Purpose	Example	Estimated cost
Spring scale 50 and 100 g)	Spring scales for weighing mice, often multiple capacities are needed to get accurate measurements but account for larger adults as well.	Pesola Lightline spring scales (Forestry Suppliers SKU 93052)	$35.95
Plastic bag	A plastic bag can be used as an anesthetization chamber, holding chamber, or observation chamber.	Hefty Jumbo storage bags (2.5 gal)	$13.41/45 bags
Masking or laboratory tape	Taping off teeth of the spring scale clasp will allow weighing of anesthetized mice from the tail.	Highland 1″ masking tape, (Staples)	$3.41
Ruler (mm markings)	For measuring body regions of trap captures.	Staples 6″ stainless steel ruler (Staples)	$3.29
Dissecting scissors	Cutting gauze and filter paper, and other miscellaneous cutting.	Dissecting scissors straight point, (Bioquip Prodcuts SKU 4713)	$5.46

### Determination of Age, Sex, and Reproductive Status

Trapping using Sherman traps unfortunately biases successful captures toward more mature mice that weigh enough to depress the trigger and be captured. In Connecticut, only 21 of 6,528 captured *P. leucopus* (0.32%) were <10 g (S. C. Williams, unpublished data). Age determination in captured mice can be challenging. Juvenile mice tend to have less mass and a gray pelage while subadult and adult *Peromyscus* tend to have greater mass and brown pelage. In Connecticut, it is estimated that *P. leucopus* <12 g are juveniles, 13–19 g are subadult, and >20 g are adults.

**Fig. 12.**
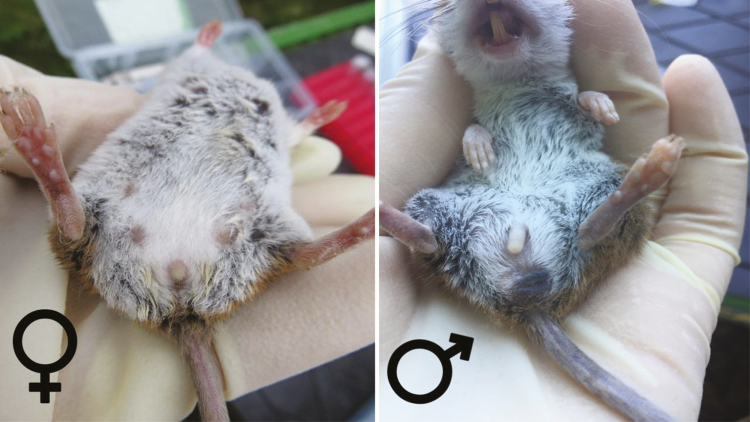
Female (left) and male (right) *Peromyscus* spp. Photos courtesy E. T. Machtinger.

Sex of captured animals can be determined by inspecting the distance between the animal’s genital region and anus. In females, the vagina is in relatively close proximity to the anus (within 0.6 cm for adults) while the testicles of male mice are further anterior (>1.0 cm) (Fig. 12). The presence of nipples is an obvious sign the mouse is female. But while male mice do not have nipples, visually undetected nipples does not mean they are not there. Use a combination of nipple presence/absence and proximity of genitals to the anus to make a final determination on sex of the individual. Reference diagrams can be found in Gurnell and Flowerdew (2019).

Pronounced nipples often indicate a lactating female and a swollen vagina can indicate that a female recently gave birth to a litter. Pregnant females are very obvious. Such reproductive clues should be recorded on your data sheet. On occasion, females give birth in traps. Whether or not to handle the female is at the researcher’s discretion, but the offspring should be released to the same trap location in a makeshift bed of leaves or other vegetative material. The adult female will likely flee when released but will likely tend to her offspring after researchers vacate the area.

### Marking Animals

Marking captured animals is essential for future identification and data management (Table 11). Past research efforts used toe clipping to mark animals, but this technique is falling out of favor due to humane concerns. Another option for permanently marking individuals is the use of passive integrated transponder (PIT) tags placed subcutaneously under the skin. PIT tags are expensive, and it is unknown by visually inspecting an individual whether it has been captured previously. In addition, research has shown that a higher percentage of PIT tags were lost in recaptured deer mice than were ear tags (Kuenzi et al. 2005).

**Table 11.** *Peromyscus* spp. marking supplies including examples and estimated cost

Item	Purpose	Example	Estimated cost
Ear tags	Necessary for individual marking of trap captures for population studies.	Laboratory Tags: Style 1005-1 (National Band and Tag Company) Ear tags, mouse size (Stoelting, Co.)	Laboratory Tags: $16.00/100 tags Stoelting Tags: $190.00/100 tags
Ear tag applicator	Necessary for ear tag application.	Laboratory Tags: National Band and Tag Company applicator Stoelting Tags: Stoelting applicator	Laboratory tag applicator: $29.40 Stoelting tag applicator: $595.00
Superfine forceps	Forceps are used to place tags and tag backs on applicator, if required (i.e., Stoelting tags).	Superfine forceps (Bioquip Products SKU 4524)	$23.27

**Fig. 13.**
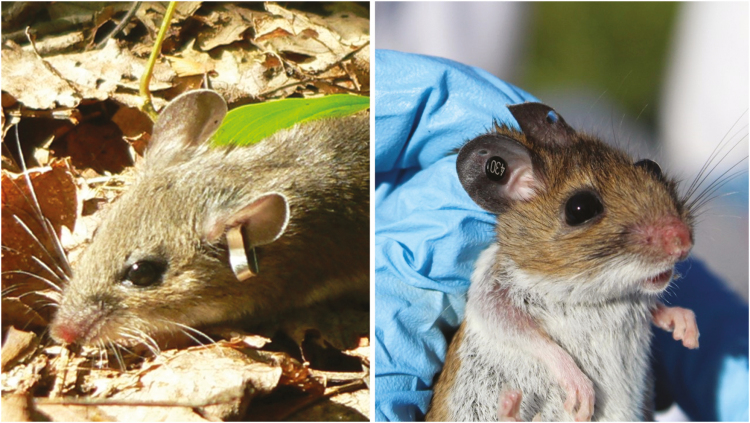
Ear tags for individual identification are easy to apply and can either be standard laboratory mouse tags (left) or Stoelting, Co. tags (right). Photos courtesy Scott Williams and Taylor Miller.

The most preferred, simple, and cost-effective method is ear-tagging. There are two types of ear tags. The first and more common are 1-cm mouse ear tags (Style 1005-1, National Band and Tag Co., Newport, KY). These have the advantage of being fairly economical, but because there is a hoop created by this tag (Fig. 13), there is a ~8% loss rate (Kuenzi et al. 2005). Tags of this type with stamped numbers are preferred over laser-etched as mice living in stone walls can render etched numbers unreadable in a matter of days or weeks. The second type of ear tag made by Stoelting, Co. (Wood Dale, IL) costs seven times more than the laboratory ear tags but has a loss rate of almost 0% (E. T. Machtinger, unpublished data) and are available in multiple colors which can be useful for data organization.

Application of either style of tag is similar. While under sedation, tags should be placed deep within the cartilaginous region of the ear to minimize the chances of it being ripped out (Fig. 13), but this is less necessary with the Stoelting tags. Because there is some risk of tag removal regardless of type, it is good practice to place tags in the same location of the same ear on each mouse, so if it does happen, you can determine that it was a previously tagged individual. Process of elimination using other recapture data, sex, mass, and body measurements can be used and often the animal number can be identified and assigned a new ear tag.

Some research has shown that ear tagged mice tend to have higher tick burdens (Ostfeld et al. 1993). If this is not acceptable for your research protocols, captured mouse ears, toes, or tails can be tattooed with different dot and color configurations to identify individuals (Chen et al. 2016) or Muromachi animal markers (Muromachi Kikai Co., Tokyo, Japan) can be used to dye pelage and last 40–50 d in the field (Haines et al. 2018).

### Tick Sampling and Identification

**Fig. 14.**
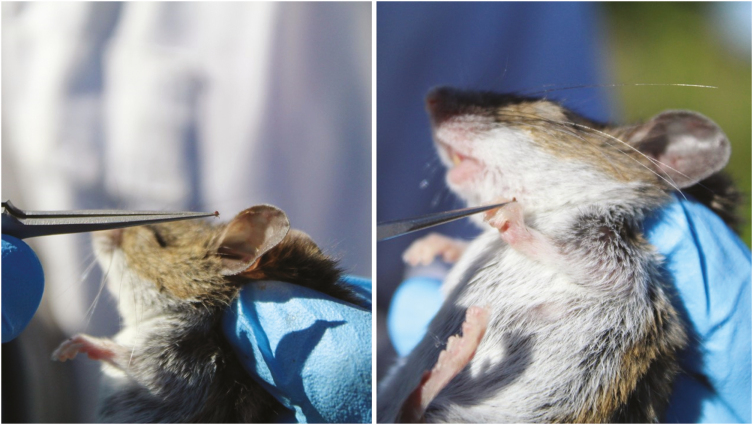
Ticks tend to congregate on the head of *Peromyscus* spp. including the ears (left) and eyes but can also be found on other areas of the body like the feet (right). Photo courtesy Taylor Miller.

The majority of visible ticks will be found on the ears and face of captured mice (Fig. 14; Ostfeld et al. 1993). Counts of visible ticks in this area will permit comparison of tick burdens between captured individuals but will be an obvious underestimate of total tick burden/mouse. If absolute tick abundance is required, the tick drop-off method by which mice are suspended in a mesh cage above water for tick drop-off can be used (Schmidt et al. 1999).

In the interest of saving time, ticks can be sampled from the head and ears using fine point, high-precision forceps and placed in a sampling container with 70–80% ethyl alcohol or another suitable preservative and returned to the lab for identification (Table 12). Care needs to be taken when sampling ticks as larvae can be easily destroyed by forceps. Once in the laboratory, parasitizing ticks can be examined under a stereomicroscope to identify them to the stage and species using reference material and an identification key (e.g., Clifford et al. 1961, Keirans and Litwak 1989, Keirans and Durden 1998).

**Table 12.** Tick collection from *Peromyscus* spp. supplies including examples and estimated cost

Item	Purpose	Example	Estimated cost
Superfine forceps	Superfine forceps are required for both nymphs and larvae, even slightly bent tips will interfere with tick recovery	Superfine forceps (Bioquip Products SKU 4524)	$23.27
Microcentrifuge or cryovials	Storage of samples	Fisherbrand Microcentrifuge tubes (Fisher Scientific SKU 05-408-137)	$63.70/500 vials
Ethanol	Preservative for tick and ear biopsy samples	Koptec V1001, (VWR International)	Varies
Vial storage box	Organization and long-term storage of samples in vials	Storage box (Fisher Scientific SKU 03-395-465)	$12.36

### How Should I Handle My Data?

One trapper should be the dedicated data recorder and should be someone with good communication skills. Hardcopy data sheets should be preprinted on Rite-in-the-Rain (JL Darling, LLC, Tacoma, WA) paper as it holds up better in wet and field conditions generally (Table 13). Data sheets can be protected and stored in a ‘field desk’ or a clipboard with storage. It is advisable to have a method of photographic record both for future study reference but also if there are anomalies or nontargets that cannot be identified. Example datasheets can be found in Mills et al. (1995).

**Table 13.** Central processing station and *Peromyscus* spp. trapping efficiency supplies including examples and estimated cost

Item	Purpose	Example	Estimated cost
Metal priming paint	Priming pant can be used to permanently distinguish the front from the back of the trap for proper orientation.	Ze-Vo Metal Wizard high gloss black coating (Home Depot)	$7.53
Folding table	Tables or tailgates can be used to process trap captures. Each allows for proper set up of supplies to streamline processing which reduces animal stress.	Enduro 5ft folding table (Dick’s Sporting Goods)	$49.99
Portable chairs	Chairs or camp stools are ideal if working from a table. Sitting allows for balanced and streamlined processing which reduces animal stress.	Field and Stream camp chair (Dick’s Sporting Goods)	$19.99
Folding canopy	Canopy cover can be extremely beneficial both when processing in precipitation and during sunny and hot days.	E-Z Up 10 × 10 Vista canopy (Dick’s Sporting Goods)	$149.99
Headlamp	Headlamps are useful when picking up or setting traps in the woods if the sun is just setting or rising.	Petzl PIXA 3 Headlamp (Forestry Suppliers SKU 217590)	$79.95
2-way radio	2-way radios can be useful for team communication when setting and picking up traps.	Motorola Talkabout T480 Rechargeable 2-way radio (Forestry Suppliers, SKU 29340)	$64.95
Field guide	Identification of non-target species is often necessary.	Peterson Field Guide of North American Mammals	$16.35
Storage and transport bins	Plastic bins with locking lids are helpful when transporting processing kits, especially if kits are going to different trapping locations.	Various	Various
Supply organization bins	Organizing supplies into divided bins can help expedite cleanup, restocking, and trap processing.	Sterilite Large Divided Case (Home Depot SKU 1001258803)	$6.98

Data to be collected should be in a logical sequence: date, location, trap #, tag #, species, sex, weights, and body measurements, and columns for other data such as number of ticks and a comment column for any additional data. The processor should call out data in the same sequence as they progress through their handling routine with each captured animal. However, it is imperative that all researchers processing animals are on the same page to ensure that the number of the tag that marks the animal is the same number on the data sheet which is the same number recorded on any samples taken from that individual. This is straightforward early in the season when captures follow a logical numeric sequence but can get more difficult with higher numbers of captures and recaptures. The processor and recorder should initial or sign each data sheet, so if questions arise, they can be consulted. When returning to the laboratory in the afternoon, data should be immediately entered into a spreadsheet program and completed hardcopy data sheets should be properly stored in the laboratory in either a binder or filing system for future reference.

### How Should I Handle Nontarget Captures?

Nontarget animal captures are a possibility during any live trapping study. If a nontarget is identified, it should be released at the location of capture. However, most IACUC reports will require a list of animals captured, so a record of capture should be made. To release nontargets, the hinge pin of the trap can be removed, so the trap can be opened without exposing the trapper to the nontarget as it escapes. Nontarget species that could potentially be captured should be addressed prior to trapping so trappers can be trained on how to handle this situation. To facilitate identification, a photograph of the nontarget could be taken, or a photo identification sheet provided of potential species in the trapping area.

### Are There Any Ways to Make My Life Easier During Trapping?

A plastic legal-size storage bin or crate without a solid bottom to facilitate drainage works well for transporting a dozen empty traps at a time into the field or occupied traps back to the central processing location. Keeping traps in the same orientation minimizes time when preparing traps; i.e., a bait can be easily applied to the roof of the trap through the back door of all dozen traps if oriented properly. To quickly determine which is the front and rear of traps, a small application of metal priming paint can be applied to the front door of each trap at the beginning of the field season. After baiting, traps can be flipped such that front doors are facing up and all doors opened to facilitate placing baited traps out in the field.

For central processing locations (Table 14), a folding table works well and can be carried into the field (Fig. 2). If using a vehicle like a pickup truck, a camping table made for mounting in a trailer hitch receiver can come in very handy. A chair is a must for the researcher handling animals to ensure proper sampling and handling with minimal distress. Chairs are optional for researchers gathering data or assisting with equipment. A portable, folding canopy can be very useful if unexpected rain showers pop up; it can be set up over field processing tables or right over the bed and tailgate of a pickup truck to permit researchers, animals, equipment, and data sheets to remain dry. This type of canopy can be helpful while cleaning traps on rainy days as well.

**Table 14.** Management of field data supplies including examples and estimated cost

Item	Purpose	Example	Estimated cost
Water resistant paper	Protection of data from dew, humidity, and rain	Rite-in-the-Rain paper (JC Darling LLC)	$14.95/50 sheets
Field desk	Storage and protection of datasheets in the field and protection from weather	Rite-in-the-Rain weatherproof field desk (JC Darling LLC)	$16.98
Binder or files	Storage of datasheets in the laboratory	Various	Various
Camera	Documentation of non-targets, atypical situations, or other phenomenon.	Various	Various
Pens	Writing on datasheets and vials	PigmaMicron archival ink pen	$3.79

## Conclusions

What has been presented is a practical guide to trapping *Peromyscus* spp. for tick and tick-borne disease surveillance. As with other scientific skills, trapping rodents is often taught by mentors or is self-taught. The goal is that the information provided herein gives a solid background to the scope of a trapping project and the necessary training, planning, and equipment for success so that research is not limited by perceived knowledge barriers. Additional resources for more in-depth, complicated, or mixed-species trapping can be found in Additional Resources.

## Additional Resources

The following is a list of supplemental reference materials that may provide additional information on trapping small mammals, or supplement the sections provided here with greater depth of explanation or alternative strategies.

**Bookhout, T. A. 1994.** Research and Management techniques for wildlife habitats, 5^th^ ed. The Wildlife Society, Bethesda, MD. 

**(CDC) Centers for Disease Control and Prevention. 2019.** Hanta Virus. https://www.cdc.gov/hantavirus/index.html [accessed 29 Jan 2020].

**(EPA) Environmental Protection Agency. 2019. ** Find the repellent that is right for you https://www.epa.gov/insect-repellents/find-repellent-right-you#search%20tool [Accessed 30 Jan 2020] 

**Duke University. 2015. ** Fieldwork safety. Durham, NC https://www.safety.duke.edu/sites/default/files/I_8FieldworkSafety.pdf [accessed 5 Feb 2020]

**Gurnell, J. and J. R. Flowerdew. 2019.** Live trapping small mammals: A practical guide, 5th ed. The Mammal Society, London, England

**Hoffmann, A., J. Decher, F. Rovero, J. Schaer, C. Voigt, and G. Wibbelt. 2010.** Field methods and techniques for monitoring mammals, pp. 482–529. In J. Eymann, J. Degreef, C. Häuser, J. C. Monje, Y. Samyn and D. VandenSpiegel (eds.), Manual on Field Recording Techniques and Protocols for All Taxa Biodiversity Inventories. Vol. 8, part 2. ABC Taxa. http://www.abctaxa.be/volumes/volume-8-manual-atbi/Part2_low_resolution.pdf

**Jones, C., W. J. McShea, M. J. Conroy, and T. H. Kunz. 1996.** Capturing Mammals, pp. 115-155. In D.E. Wilson, F. R. Cole, J. D. Nichols, R. Rudran, and M. S. Foster (eds), Measuring and monitoring biological diversity. Standard methods for mammals. Smithsonian Institution Press, Washington and London.

**Mills, J. N., J. E. Childs, T. G. Ksiazek, C. J. Peters, and W. M. Velleca. 1995.** Methods for trapping and sampling small mammals for virologic testing. Centers for Disease Control and Prevention U.S. Department of Health and Human Services. Atlanta, GA https://stacks.cdc.gov/view/cdc/11507

**Sikes, R. S. and the Animal Care and Use Committee of the American Society of Mammalogists. 2016.** 2016 Guidelines of the American Society of Mammalogists for the use of wild mammals in research and education. Journal of Mammalogy 97:663 – 688.

**Silvy, N. J. 2012.** The wildlife techniques manual, 7th ed. Johns Hopkins University Press, Baltimore, MD. 

**Thibault, K. 2016.** TOS protocol and procedure: Small mammal sampling. National Ecological Observatory Network. https://data.neonscience.org/documents/10179/1883155/NEON.DOC.000481vG/a7614605-ba6a-4f11-ad18-301a9a42aeec [Accessed 4 Feb 2020]
